# No support that early selective dorsal rhizotomy increase frequency of scoliosis and spinal pain – a longitudinal population-based register study from four to 25 years of age

**DOI:** 10.1186/s12891-020-03782-5

**Published:** 2020-11-27

**Authors:** Annika Lundkvist Josenby, Lena Westbom

**Affiliations:** 1 Children’s Hospital, Skåne University Hospital, Lund, Sweden; 2 Faculty of Medicine, Department of Health Sciences, Lund University, Lund, Sweden; 3 Faculty of Medicine, Department of Clinical Sciences Lund, Paediatrics, Lund University, Lund, Sweden

**Keywords:** Cerebral palsy, Selective dorsal rhizotomy, Complications, Scoliosis, Spinal pain, Population-based, Controlled registry study

## Abstract

**Abstract:**

Spasticity interfering with gross motor development in cerebral palsy (CP) can be reduced with selective dorsal rhizotomy (SDR). Although reported, it is unknown if SDR surgery causes later spine problems. Using CP-registry data from a geographically defined population, the objectives were to compare frequency and time to scoliosis, and spinal pain up to adult age after SDR-surgery or not in all with same medical history, functional abilities, CP-subtype and level of spasticity at 4 years of age. Variables associated with scoliosis at 20 years of age were explored.

**Method:**

In the total population with CP spastic diplegia in Skåne and Blekinge, born 1990–2006, 149 individuals had moderate to severe spasticity and no medical contraindications against SDR at 4 years of age and were included; 36 had undergone SDR at a median age of 4.0 years (range 2.5–6.6 years), and 113 had not.

Frequency of scoliosis and age when scoliosis was identified, and frequency of spinal pain at 10, 15, 20 and 25 years of age were analysed using Kaplan-Meier survival curves and Fisher’s exact test. Multivariable logistic regression was performed to identify variables to explain scoliosis at 20 years of age. Gross Motor Function Classification System (GMFCS) levels at 4 years of age were used for stratification.

**Result:**

Frequency of scoliosis did not significantly differ between groups having had early SDR surgery or not. In GMFCS IV, the SDR group had later onset and lower occurrence of scoliosis (*p* = 0.004). Frequency of spinal pain did not differ between the groups (*p*- levels > 0.28). GMFCS level was the background variable that in the logistic regression explained scoliosis at 20 years of age.

**Conclusion:**

Frequency of back pain and scoliosis in adulthood after early SDR are mainly part of the natural development with age, and not a surgery complication.

**Supplementary Information:**

The online version contains supplementary material available at 10.1186/s12891-020-03782-5.

## Introduction

Selective dorsal rhizotomy (SDR) is a neurosurgical procedure for children with spastic diplegic cerebral palsy (CP) that permanently reduces spasticity in the lower limbs by cutting parts of lumbosacral rootlets at spinal levels L2-S2. SDR is always combined with physical therapy, and it is mainly used in young children to improve future functional skills [1]. Neurosurgeons use either multilevel or single level laminotomy surgery to access the rootlets. As the intervention includes surgery to the spine and spinal nerve roots, there is a hypothetical risk that SDR will cause spinal deformities and pain may develop. The single level laminotomy technique was developed for the advantages of decreased time for surgery, postoperative pain and to minimize the risk of progressive lumbar instability [2]. In a short-term follow-up, no significant differences between the two techniques has been found [3].

After SDR, scoliosis was reported to be the most common spinal deformity occurring at a weighted mean incidence of 31.6% [4], and Cobb angles ≥20° have been reported at 3–9% 2.8–11.6 years after SDR [5–7]. In another study of children with pre-existing scoliosis prior to SDR, 5% improved Cobb angle, 70% were unchanged, and 25% had worsened at a mean follow-up time of 4.3 years [8]. Peter et al. and Langerak et al. followed the same ambulatory cohort over time, and at the five-year follow-up after SDR, scoliosis of ≥10° was found in 16% at 4.5 years and 57% at 21 years follow-up, of which 7% had Cobb angles of ≥30°. None of the participants had scoliosis prior to SDR [9, 10].

In follow-up studies from 3.6 to 21.4 years after SDR, hyperlordosis was found to increase compared to baseline in 10–50%, and a hyperkyphosis was reported in 1–9%. Individuals who were walking with and without walking aids generally had, postoperatively, larger lumbar lordosis [6, 8, 9, 11–13].

Spondylolisthesis was uncommon preoperatively and postoperative prevalence ranged from 2 to 27%, mainly minor to grade 1 slippage [6, 7, 9, 11–13]. In a 20–28- year follow-up after SDR, Park et al. found 31% with scoliosis and other spinal problems [14].

On the other hand, persons with CP are generally more frequently affected by spinal abnormalities, particularly scoliosis, than the general population [15].

Follow-up studies of adult individuals walking with or without devices, who had undergone SDR during the preschool years to early school age, show occurrence of spinal pain in 17–23% [9] and pain in spine and lower limbs in 28% [14]. In a follow-up with shorter time frame, spinal pain was reported at a lower level, 5% in an self- ambulant cohort 5.8 years post SDR [13].

In previous population based studies, excluding persons treated with SDR, Kaplan–Meier survival estimates based on the results of the clinical examination showed that scoliosis was seen in younger ages in children with higher GMFCS levels meaning more severe functional limitations. The incidence of scoliosis increased with age and GMFCS level. The incidence of spinal pain in CP was shown to increase with increasing scoliosis severity, and scoliosis incidence increased with severity of gross motor dysfunction [16, 17].

Today it is unknown whether the above figures regarding spine problems after SDR actually differ from the situation in persons with the corresponding type and severity of CP who are not treated with SDR. In a recently published review of spinal deformities after SDR, the authors suggested that the occurrence of spinal deformities were most likely not different, or only minimally higher after SDR, than in the natural history of CP, girls were more likely to develop scoliosis, and that the deformity develops over a long time, but the evidence base was weak [4].

Incidence of spinal deformity and pain after SDR is an important complication to consider, when deciding whether to proceed with the procedure or not, as spinal deformity can have a major impact on quality of life [18]. There is a need for longitudinal follow-up studies of spinal pain and deformities in individuals undergoing SDR, taking the natural development in cerebral palsy into account.

The objectives of the present study were to compare frequency and age when scoliosis was diagnosed, and frequency of spinal pain in individuals who had undergone SDR, and in a control group with same CP-type, medical history, body structure and functions at 4 years of age, living in the same geographically defined total population, followed from birth to adult age in the same structured program and registry. Another objective was to explore if any of the variables SDR surgery, sex, spasticity and GMFCS level at 4 years of age, was associated with the presence of scoliosis at 20 years of age in this population.

## Methods

A retrospective population based controlled long-term outcome study using data from the Swedish cerebral palsy follow-up program (CPUP) including prospectively registered results from repeated, most often yearly, clinical assessments in children, adolescents and adults with CP, living in a defined geographical area in southern Sweden [19].

### Setting

#### CPUP

The secondary prevention follow-up program in cerebral palsy (CPUP) was started in the regions of Skåne and Blekinge in 1994, as a cooperation project between the child orthopaedic and paediatric neurology departments in tertiary care level, and the local child (re) habilitation services and orthopaedic units in the area. The aims were to prevent hip dislocations and severe contractures, increase cooperation, and generate more knowledge about CP, especially the natural history and long-term results of different treatments. New treatment modalities at the time were intrathecal baclofen (ITB) and SDR [20].

The original program was based on structured follow-up and evaluation of hip radiographs and repeated, structured, often annual physiotherapy (PT) assessments [19]. To identify all children with CP in the population, regularly repeated inventories were performed in Skåne and Blekinge to find the youngest children, not yet invited to the program, and later on, after 4 years of age to decide on CP diagnosis and subtype for children in the program [21, 22].

Data for this study was retrieved from the CPUP demographic patient forms, neuropediatric, PT assessments, and operations forms from 1994 to 2018, validated and completed by scrutinizing medical records covering most of the health care systems in the two regions during the study period. Demographic data was checked against the Swedish population registry every year, including dates for births, deaths, moving in and out of the area.

#### SDR

Some of the children with BSCP and moderate to severe spasticity, were referred from their local (re-)habilitation units in the Skåne-Blekinge area for evaluation regarding spasticity management, and if selected for SDR, the surgical intervention and the immediate postoperative rehabilitation took place at the Skåne University Hospital in Lund. Indications for SDR were spastic cerebral palsy with more involvement in the legs than the arms, pure spasticity without dystonia and ataxia, spasticity interfering with functional development, enough muscular control and strength to reach the individual functional goals, and access to regular postoperative physical therapy and orthotic services. Individual goals were set together with family, local (re) habilitation unit and the spasticity team [23]. During the last two decades Magnetic Resonance Imaging (MRI) of the brain has been recommended at about 18–24 months of age in children with CP, which, although mostly seen clinically as dyskinesia or ataxia, has added involvement of thalamus/basal ganglia or cerebellum to the contraindications for SDR.

### Participants

#### Population

The prevalence of CP in the study area was 2.7/1000 children 4–11 years of age as of Jan 1st, 2002, of which 38% was spastic diplegia [21]. The present study was based on all persons born 1990–2006, with CP spastic diplegia, who lived in Skåne-Blekinge for at least 2 years during the years 1994–2015; there were 267 persons participating in the CPUP-program and seven (2.5%) who did not. Demographic, medical and functional characteristics of the population with CP spastic diplegia in the study area, and the distribution of these characteristics in the study groups are presented in Table 1.

**Table 1:** Characteristic of all participants in CPUP with CP spastic diplegia, *N* = 267

	SDR group ***n*** = 36(%)	No contraindication - no SDR		Excluded with contraindication for SDR *n* = 55 (%)
		Non-SDR group ***n*** = 113 (%)	Excluded mild spasticity *n* = 63 (%)	
Year of birth*				
1990–1993	13 (36)	31 (27)	12 (19)	13 (23,5)
1994–1997	14 (39)	27 (24)	22 (35)	9 (16.5)
1998–2001	7 (19.5)	27 (24)	13 (21)	14 (25.5)
2002–2006	2 (5.5)	28 (25)	16 (25)	19 (34.5)
**Birth country other than Sweden**	6 (17)	12 (11)	11 (16)	6 (11)
**Sex**				
Male	25 (69)	60 (53)	37 (60)	41 (74.5)
Female	11 (31)	53 (47)	26 (40)	14 (25.5)
**Gestational Age (GA)**				
GA < 26 weeks	2 (6)	7 (6)	8 (12.5)	1 (2)
GA 26–27 weeks	3 (8)	17 (15)	5 (8)	1 (2)
GA 28–31 weeks	16 (44.5)	31 (27.5)	13 (21)	2 (3,5)
GA 32–36 weeks	8 (22)	24 (21)	19 (30.5)	13 (23.5)
GA > 36 weeks	7 (19.5)	29 (25.5)	12 (19)	35 (63.5)
Unknown	0	5 (4)	6 (9.5)	3 (5.5)
**Birth weight**				
< 1000 g	3 (8)	23 (20)	11 (17.5)	2 (4)
1000–1499 g	8 (22)	21 (19)	11 (17.5)	1 (2)
1500–2499 g	15 (42)	23 (20)	14 (22)	7 (12.5)
2500–4999 g	9 (25)	26 (23)	14 (22)	27 (49)
Unknown	1 (3)	20 (18)	13 (21)	18 (32.5)
**Multiple pregnancy**	5 (14)	25 (22)	6 (10)	2 (4)
**Severe asphyxia > GA 34 weeks**	1 (3)	0	0	15 (27)
**Post-neonatal CP**	0	0	0	7 (13)
**CNS imaging (dominating pattern)**				
Maldevelopments	0	0	8 (13)	26 (47.5)
Predominant white matter injury (periventricular)	17 (47)	74 (65.5)	33 (52)	16 (29)
Basal ganglia/thalamus lesions	0	0	0	0
Cortical/subcortical grey matter lesions	1 (3)	4 (3.5)	2 (3)	5 (9)
Normal	2 (6)	9 (8)	4 (6)	5 (9)
No CNS imaging	16 (44)	26 (23)	16 (26)	3 (5.5)
**Shunted hydrocephalus**	4 (11)	15 (13)	13 (21)	20 (36)
**Epilepsy**	6 (17)	27 (24)	12 (19)	23 (42)
**Intellectual disability**				
None or mild (IQ > 50)	34 (94)	93 (82)	57 (90)	36 (65)
Moderate or severe (IQ < 50)	2 (6)	20 (18)	6 (10)	18 (33)
Missing info	0	0	0	1 (2)
**Severe visual disability/blindness**	4 (11)	20 (18)	7 (11)	10 (18)
**GMFCS levels at 4 years**				
I	2 (5.5)	43 (38)	44 (70)	16 (30)
II	11 (30.5)	18 (16)	6 (10)	12 (22)
III	11 (30.5)	20 (18)	8 (12)	15 (28)
IV	12 (33.5)	23 (20)	5 (8)	4 (7)
V	0	9 (8)	0	7 (13)
Missing info	0	0	0	1
Spasticity level at 4 years***				
Mild	0	0	50 (79)	13 (24)
Moderate	12 (33)	83 (73)	0	29 (53)
Severe	24 (67)	30 (27)	0	10 (18)
Missing info	0	0	13 (21)	3 (5)

Legend: *CPUP* The Swedish national secondary prevention follow-up program in cerebral palsy, *SDR* Selective Dorsal Rhizotomy, *CP* Cerebral Palsy, *GA* Gestational Age, *ITB* Intrathecal Baclofen, *GMFCS* Gross Motor Function Classification System, *CNS* Central Nervous System, *IQ* Tested or estimated cognitive level. Differences between the SDR group and the control group n.s., except regarding birth year cohorts* (*p* < 0.05) and spasticity levels*** (*p* < 0.001)

#### SDR group

**Fig. 1:**
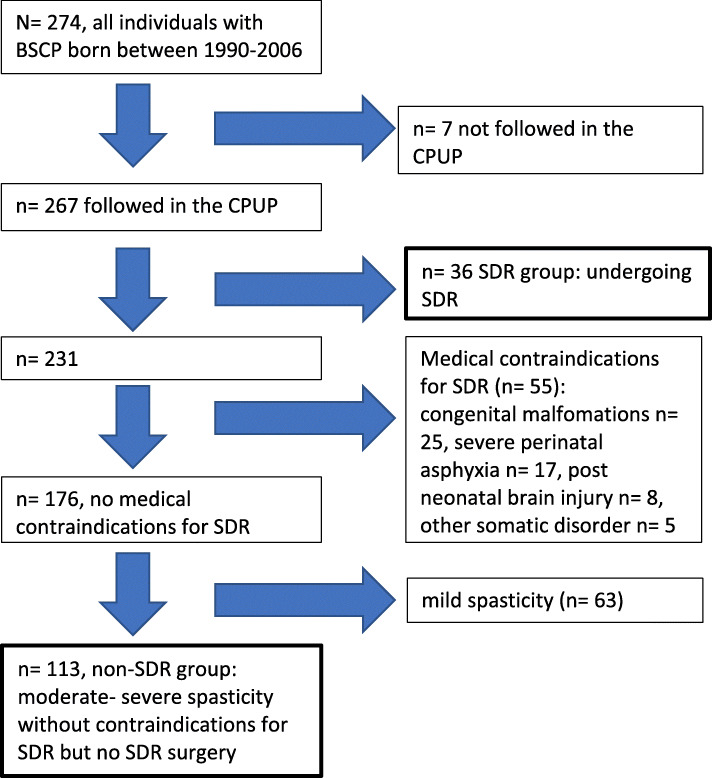
Flow chart illustrating inclusion and exclusion of individuals in SDR group and control group

Of the 267 CPUP participants with spastic diplegia, there were 36 persons who had undergone SDR surgery at a median age of 4.0 years (range 2.5–6.6 years). They were followed to a median age of 22.3 years (range 11.3–27.2); 30 of the 36 SDR-operated persons had CPUP assessments at 20 years of age; eight had reached and been assessed at 25 years of age at the end of the study, January 1st, 2018 (Table 1) (Fig. 1).

#### Excluded

Of the remaining 231 persons with CP spastic diplegia in the study population, there were 55 persons with medical contraindications to SDR; congenital malformations or syndromes in 25 persons, severe perinatal asphyxia in 17, post-neonatal brain injuries in eight, and five persons had other severe somatic disorders.

Classification of spasticity level at 4 years of age, as described below, was mild in 63 persons, without any indication for SDR, and they were therefore excluded from the comparison (Table 1) (Fig. 1).

#### Control group

The remaining 113 persons with CP spastic diplegia, moderate-severe spasticity level and no medical contraindications against SDR constituted the control group (Table 1) (Fig. 1). As of January 1, 2018, this natural history control group was followed to a median of 19.6 years of age (range 8.9–27.3 years).

Six children treated with ITB were excluded from the control group after the ITB-operation at 5, 6, 11, 14, 16 and 18 years of age respectively. In addition, two persons in the control group showed to be too young (< 8 years of age) at the latest assessment and were not included in the comparison. In the control group, 54/113 had reached and had assessments at 20 years of age, and 16/113 at 25 years.

### Definitions and classifications

*Cerebral palsy* (CP) was defined according to Much et al. 1992 [24] and the Surveillance of Cerebral Palsy in Europe (SCPE) [25].

#### CP subtype

Subtypes were defined by the dominating neurological symptom between four and 7 years of age. All patients in this study had Bilateral Spastic CP (BSCP) as defined by the SCPE, and all also fulfilled the definition of the subtype spastic diplegia in the Hagberg classification [21, 25]. CP spastic diplegia includes all five GMFCS-levels, as long as there is greater involvement of the legs than of the arms, and can be asymmetrical, with more involvement of either left or right side of the body [26].

*Brain morphology* was classified based on the dominating pattern on brain imaging according to the SCPE [27].

*Severe asphyxia* was classified based on low Apgar scores (< 4 at 5 min), clinical and EEG-verified seizure activities within the first 72 h after birth.

*Epilepsy* was defined as having had at least two unprovoked seizures after the neonatal period.

*Intellectual disability* was assessed at about 4 years of age. Only parts of the cohort were formally IQ-tested at that age, and only moderate to severe intellectual disability was defined as intellectual disability in this paper. For the majority of the children, this is a clinical estimation with inherent uncertainty.

The *Gross Motor Function Classification System (GMFCS)* [28] was used to classify gross motor function at each PT assessment in CPUP. The GMFCS level classified at the first assessment after the child’s fourth birthday, or pre-operatively if earlier SDR surgery, was used. The GMFCS was used for classification of gross motor function in the CPUP from the year 1995 by some physiotherapists as part of the development/testing of the classification, and from 1998, the classification was introduced for all PT assessments in CPUP and have shown good stability in previous studies in this population [29].

The GMFCS-level was classified by the child’s physiotherapist in 239 of all 267 persons with BSCP. In the oldest cohort, the GMFCS level was classified using other entered CPUP-data; the reliability of such a retrospective classification was first confirmed, see Additional file 1. The classification was performed on de-identified data with no information available regarding if the child belonged to the control or the SDR group.

*Muscle tone* was assessed according to the Bohannon Modified Ashworth Scale (MAS) [30]. MAS-scores for five muscle groups (hip-flexors, hip-adductors, knee-extensors, knee-flexors, and plantar-flexors) in both legs were summed up for each CPUP participant with BSCP, who had complete recordings from all included muscle groups at 4 years of age. The MAS scores 1 and 1+ were both counted as 1. Median MAS summation score was 7 (range 0–32, inter- quartile range 4–12).

Muscle tone assessed with MAS is closely related to GMFCS level [31]. When occasional single MAS-scores were missing in the study groups, imputation was therefore performed by using the median score for the missing specific muscle group(s) of all with completely recorded MAS scores and the same GMFCS level. Six children in the SDR group and 12 in the control group had imputed single MAS scores.

*Estimated spasticity level* at 4 years of age was classified based on muscle tone (MAS summation score) and clinical signs, such as degree of leg scissoring (none, mild, severe) in rest and in activity, and foot clonus (yes/no), as assessed by each child’s local PT according to the structured follow-up form in CPUP (http://cpup.se/in-english/manuals-and-evaluation-forms).

Spasticity level was defined as *mild* if the MAS summation score was within the first quartile 0–3, *moderate* if in the second quartile 4–6, and *severe* if summation score was 13 or higher. In the third quartile, MAS summation scores 7–12 the spasticity level was defined as *severe* if combined with severe scissoring in activity, and if not, it was classified as a *moderate* spasticity level.

*Scoliosis* was assessed by inspection by the physiotherapist and registered to be either present or not. If present, *mild:* scoliosis visible only when leaning forward with aligned pelvis, *moderate*: scoliosis visible both in leaning forward in sitting and when sitting straight, and *severe*: scoliosis, not correctable, with need of support in sitting or standing. The CPUP PT assessment of the spine has shown to have high inter-rater reliability and specificity in screening for moderate to severe scoliosis in this population [32].

In the present study, scoliosis status was dichotomized: *no scoliosis* if there was no scoliosis at examination or when a scoliosis was assessed as being correctable, and *existing scoliosis* if scoliosis was assessed by the PT as being severe or moderate and not correctable, or if the individual had been undergoing scoliosis surgery. Dates/age at first PT assessment with existing scoliosis and at scoliosis surgery were noted.

Questions about *experienced pain and localization of pain* were introduced in the PT assessments from January 2007 [33]. Presence of pain was assessed by either the person him/herself or by proxy. For children (< 18 years) pain in the spine was reported by answering yes/no and for adults (> 18 years) recalling the last 4 weeks for spinal pain that was classified as being moderate or severe. After 2016, children were also asked to recall presence of pain during the last 4 weeks.

### Statistics

Kaplan-Meier survival analyses were performed to analyze time to scoliosis in relation to the following dichotomous variables; SDR surgery (yes vs no), GMFCS level (I-II vs III-V), level of base-line spasticity (moderate vs severe) and sex (male vs female).

Kaplan Meier survival analysis was further used to compare time to scoliosis, stratifying for SDR, and severity of gross motor function limitation, defined as GMFCS level at 4 years of age. Sub-groups were created according to GMFCS levels; I-II (*n* = 74), III (*n* = 31) and IV-V (*n* = 44).

Two-sided Pearson’s Chi-Square and Fisher’s exact tests were used for comparisons between the SDR and the non- SDR group background factors (Table 1) and pain at different age, and Kappa agreement test to check validity of retrospective GMFCS-classifications (Additional file 1).

To explore which variables that influenced the development of scoliosis at 20 years of age, the first step was to perform univariable logistic regression analyses of dichotomous variables in those who had reached this age (*n* = 82). Following variables were analyzed; SDR (yes/no), GMFCS at 4 years of age (I-II vs III-V), spasticity at 4 years of age (moderate/severe) and sex (male/female).

All the before mentioned variables, except sex, were included in the model when we, as the second step, performed the multivariable logistic regression analysis. Due to the small number of participants with scoliosis in both the SDR and non-SDR group, only a small number of variables could be included in the model.

Statistical significance was set at *p* < 0.05; n.s. denotes no statistically significant difference.

IBM SPSS statistics, version 25 was used for analyses [34].

## Results

The background factors were similar in the SDR group and control group (Table 1). A higher proportion were SDR-operated in the first two birth cohorts than in the younger cohorts born from 1998 (*p* = 0.043). Another difference was a lower proportion of severe spasticity in the control group than in the SDR group (*p* = 0) (Table 1).

### Scoliosis

Only two of the SDR-operated children were in GMFCS level I, and neither had scoliosis at the end of the study. Two of the 43 in the control group GMFCS level I had scoliosis at the end of the study (n.s.). No child in GMFCS V had had SDR-surgery. Two of the seven individuals in the non-SDR group GMFCS V assessed at 20 years of age had scoliosis.

In GMFCS levels II-IV no child in either group, had scoliosis before 10 years of age. Scoliosis was less frequent and developed later in GMFCS II-III with higher level of motor function, than in GMFCS IV.

**Fig. 2:**
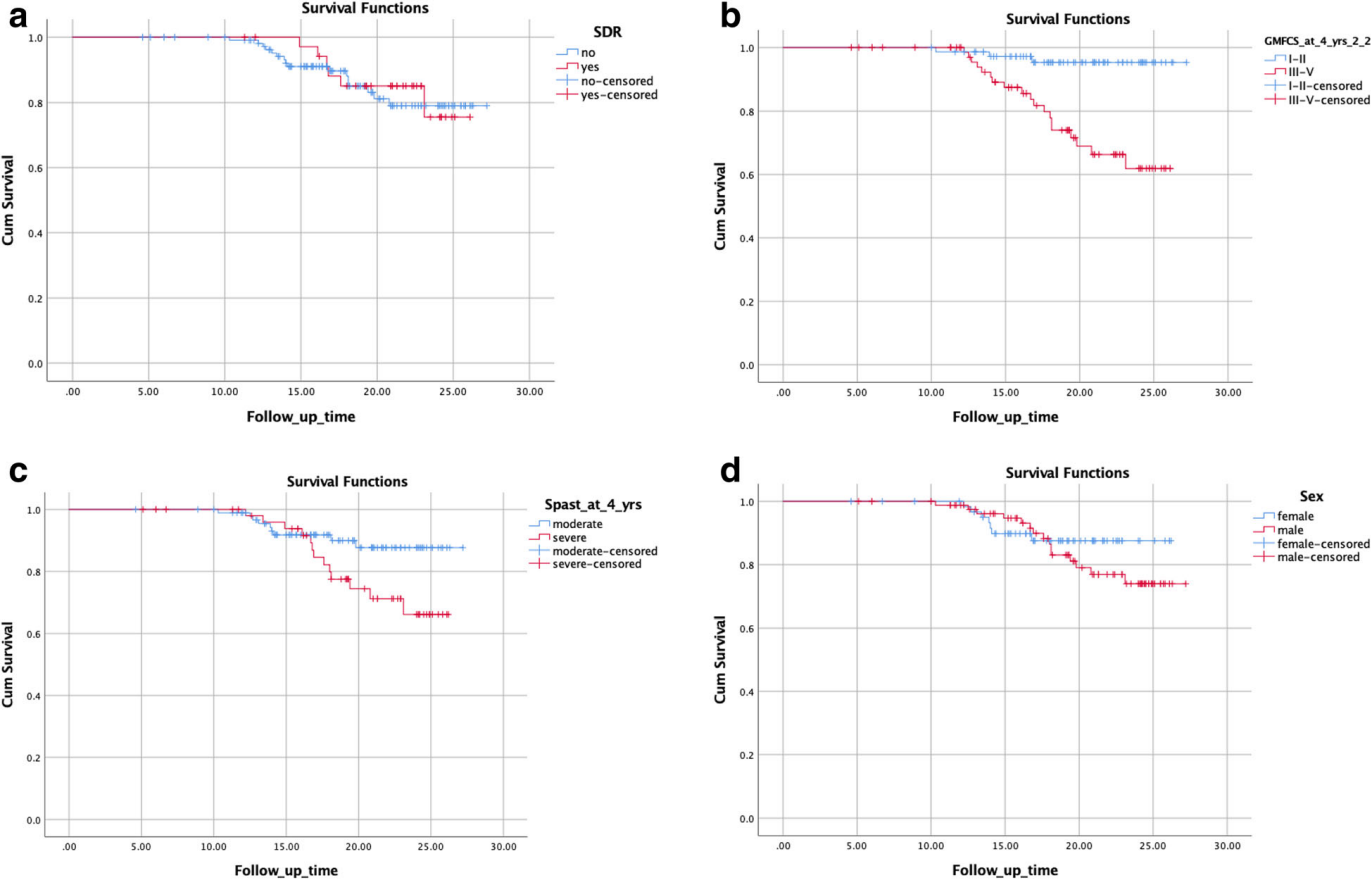
**a**. Kaplan-Meier survival curves illustrating the influence of SDR surgery (SDR vs no SDR) in the development of scoliosis (*n* = 149) *p* = 0.822. **b**. Kaplan-Meier survival curves illustrating the influence of GMFCS level at the age of 4 years in the development of scoliosis (*n* = 149) *p* < 0.001. **c**. Kaplan-Meier survival curves illustrating the influence of spasticity at the age of 4 years (moderate vs severe) in the development of scoliosis (*n* = 149) *p* = 0.045. **d**. Kaplan-Meier survival curves illustrating the influence of sex (male vs female) in the development of scoliosis (*n* = 149) *p* = 0.387

In the Kaplan-Meier analyses, the variables *GMFCS* (*p* < 0.001), and base-line s*pasticity level* showed significant differences between the two groups (*p* = 0.045) in development of scoliosis, however SDR surgery (*p* = 0.822) and sex (*p* = 0.387) did not. (Fig. 2a-d).

**Fig. 3:**
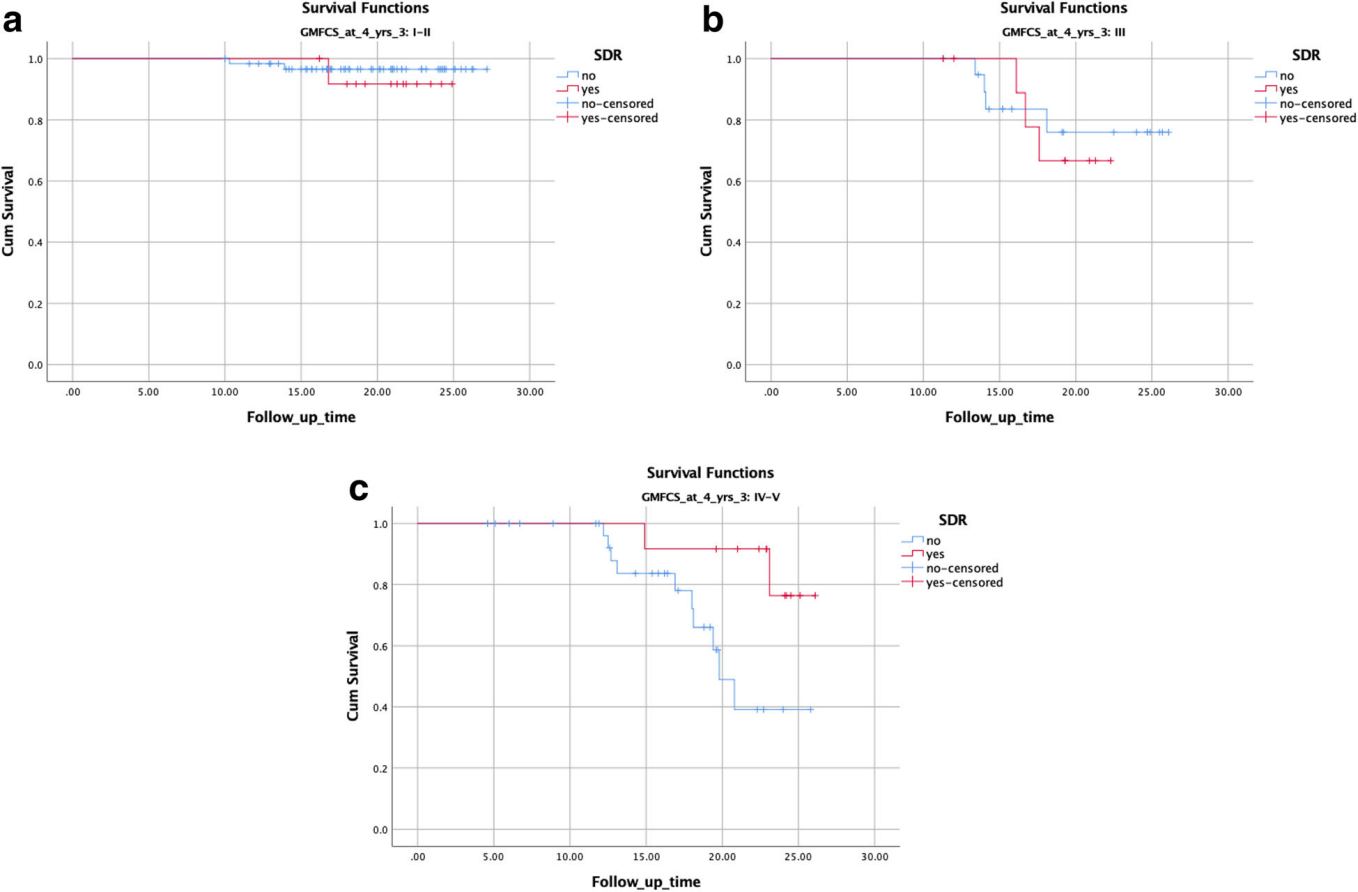
**a**. Kaplan-Meier curve illustrating age when scoliosis according to the study definition was first reported in GMFCS level I-II, *p* = 0.567. **b**. Kaplan-Meier curve illustrating age when scoliosis according to the study definition was first reported in GMFCS level III, *p* = 0.778. **c**. Kaplan-Meier curve illustrating age when scoliosis according to the study definition was first reported in GMFCS level IV-V, *p* = 0.026

Individuals in GMFCS levels IV-V undergoing SDR had less scoliosis and a later onset of scoliosis compared to the non- SDR group (*p* = 0.026) using the Kaplan-Meier analysis. In GMFCS subgroups I-II and III, the differences were not statistically significant (*p* = 0.567 and *p* = 0.778 respectively) (Fig. 3a-c).

All 12 in the SDR group GMFCS IV were followed to age 15 years, and 11 were followed to age 20 years; one SDR group participant GMFCS IV had scoliosis at age 14 years, and one at age 23 years. Eight of the 23 control group participants GMFCS IV developed a scoliosis at 12, 12, 12, 13, 16, 18, 18, and 19 years of age respectively. Six of the 14 in the control group followed to at least 15 years of age had scoliosis before end of study, and all five followed to at least their 20th birthday had scoliosis before that age (Fig. 3c).

In the univariable analysis, *GMFCS level* was the variable that significantly explained the presence of moderate- severe scoliosis at 20 years of age. In the multivariable analysis, the result was maintained (Table 2). Neither the variables *SDR surgery* nor base-line *spasticity level* made statistically significant contribution in explaining scoliosis at 20 years of age in the regression model (Table 1).

**Table 2:** Variables influencing development of scoliosis or not at 20 years of age in univariable and multivariable analyses (*n* = 82)

Variables	Univariable			Multivariable		
	OR	95% CI	p	OR	95% CI	p
SDR	0.65	0.21–2.04	0.460	0.37	0.093–1.50	0.164
GMFCS at 4 yrs	7.85	2.08–29.6	0.0023	8.54	2.01–36.4	0.004
Spasticity at 4 yrs	1.94	0.70–5.36	0.204	1.27	0.33–4.89	0.729
Sex	1.17	0.41–3.36	0.766	–	–	–

Legend: Selective dorsal rhizotomy (SDR), Gross Motor Function Classification System (GMFCS), Odds ratio (OR), Confidence interval (CI)

### Spinal pain

Patient’s reports of spinal pain at 10, 15, 20 and 25 years of age are presented in Table 3. No statistically significant difference was observed between the SDR and the control group (Table 3). Questions regarding pain were introduced in CPUP in January 2007, and therefore pain assessments at 10 and 15 years of age were missing for the oldest cohorts. Younger cohorts had not yet reached the oldest ages at data extraction.

**Table 3:** Reported spinal pain at different ages in GMFCS levels II-IV, SDR and non-SDR groups

Groups	10 years		15 years		20 years		25 years	
	Pain	No pain	Pain	No pain	Pain	No pain	Pain	No pain
SDR	1	14	5	26	6	22	4	4
Non-SDR	1	39	6	42	3	20	1	6
*p*- value	*p* = 0.475		*p* = 0.7436		*p* = 0.488		*p* = 0.282	

Legend: *GMFCS* Gross Motor Function Classification System, *SDR* Selective Dorsal Rhizotomy

Of all those in GMFCS IV who were followed to the 20 years assessment, one of 12 in the SDR and one of seven in the non-SDR group reported spinal pain; both had had scoliosis surgery more than 4 years earlier, and both had severe spinal pain, interfering with daily activities.

## Discussion

This is the first population based controlled SDR long-term outcome study. All individuals in the geographically defined population (98% participating in the CPUP registry) with a medical background and clinical expression that matched the selection criteria for SDR at baseline were included in the study [1]. Among these all having had SDR-surgery were compared to all who had not. .

Prospectively collected longitudinal follow-up data are presented for scoliosis and spinal pain from the population of children with spastic diplegic CP, where a group of individuals had undergone SDR at a young age. Neither scoliosis nor spinal pain was more prevalent during 20 years of follow-up in the SDR group, after the cauda equina multilevel surgery, compared to the control group representing the natural history in children with about the same base-line prerequisites, and with same standard of care before and during follow up. In children at GMFCS-level IV at baseline, without functional walking ability, scoliosis developed later and less often in the SDR group during the following 20 years than in the control group (Fig. 3c).

As SDR has been a challenged treatment option, few children have been referred for evaluation. This study indicates that feared complications to early SDR, such as increased frequency of back pain and scoliosis in adulthood, are mainly part of the natural development with age, and not a surgery complication. We now can inform rehabilitation professionals and parents of future SDR candidates that permanent spasticity reduction by SDR can be obtained without increased occurrence of scoliosis or spinal pain, at least until and including early adulthood. Still, spinal problems are common in cerebral palsy and should be early identified and treated in order to minimize future discomfort and pain.

### Population, registry and standard of care issues

The CPUP registry data provided information from standardized and regular assessments performed and recorded by the person’s own physiotherapist at certain ages, same procedures for all individuals, regardless of whether they had undergone SDR surgery or not [19].

All persons in the study were treated at the same public health care units; orthopedic and pediatric hospital health care departments, and the habilitation services in cooperation. Physiotherapy, occupational therapy, social and psychological support, orthoses, braces, orthopedic surgery, ITB, SDR and from 1998 botulinum toxin injections were part of standard care, and with no or very low economic costs for the patients.

Almost all children with CP spastic diplegia in the area were included. Ten children enrolled in CPUP after SDR; their preoperative GMFCS level and muscle tone was assessed and recorded in the medical records by the spasticity-team physiotherapist. All other assessments were performed and recorded in CPUP by the person’s local (re) habilitation personnel, which made it possible to study effects of SDR in this population, without any selection bias or bias regarding expectations on SDR-results.

### Natural history non-SDR group

When SDR was introduced in Lund 1993, three North American randomized controlled trials (RCTs) were underway and preliminary results were forthcoming [35]. At the time, an RCT in the Lund university hospital uptake region was considered both unethical and non-feasible due to the small population eligible. Even if the available RCTs showed promising short-term results, monitoring of long-term effects of SDR was needed. In addition to practice-based follow up [36, 37], the CPUP program/registry was planned at SDR-start, and among the aims were to follow the natural history and relation to long-term results of treatments [20]. The program was started in Skåne and Blekinge 1994 and included persons with CP born in 1990 and later, followed regularly since that time.

The CPUP registry data indicated that only few of the children who could have benefited from an SDR were referred to the spasticity clinic during early childhood, especially after treatment with botulinum toxin was introduced in 1998. It is thus likely that more children might have been recommended SDR if they had been referred to the tertiary spasticity clinic where selection for SDR was performed. For this study, we could create a group for comparison with clinical background and physical expression to match the selection criteria for SDR [38]. Some may have had features not visible in the registry data that differed from those who actually underwent SDR, such as dependence on spasticity for walking and standing, or other barriers to reach the desired functional goals with the intervention. There were also some children included in the non-SDR group who were recommended SDR by the spasticity team, but their parents did not choose the intervention.

The pediatric spasticity team members at Skåne University Hospital have had the same selection criteria and follow-up procedures since the start in 1993 [23]. Contraindications to SDR were exclusion criteria in the present study, such as mild spasticity, malformation syndromes, postneonatally acquired CP, CP due to prenatal/congenital infections, severe birth asphyxia, and dyskinetic, ataxic, unilateral spastic, or mixed CP subtypes.

Periventricular leukomalacia or hemorrhages, often in combination with premature birth, are associated with the CP subtype spastic diplegia, often suited for the SDR-intervention [1]. Such white matter brain lesions were present in the majority of both the SDR group (17/20, 85%) and the control group (74/87, 85%) who had had brain imaging (Table 1). Less CNS-imaging in the SDR group than among controls was due to the higher proportion SDR-surgery in the oldest age cohorts, before brain imaging was recommended in CP diagnostic work-up.

### Spasticity

Even if the MAS have shown weak psychometrical properties [39] it has been used by physiotherapists for assessing muscle tone in the CPUP follow-up since the start in 1994. To create study groups at baseline that correspond to the selection criteria for SDR, an estimated spasticity level classification was performed as described. The MAS is an ordinal scale and does not methodologically allow such calculations, however it estimates a clinically significant entity used for classification and not for evaluation of interventions. It makes clinical sense that a child with a high degree of muscle tone in all muscle groups will get a high summation score in contrast to a child with less tone, who may show an increase in just distal muscle groups. In our study, we added clinical signs of spasticity to the MAS summation score quartiles, such as leg scissoring at rest and activity, to classify muscle tone increase into mild, moderate and severe estimated spasticity levels. We found clear cut-offs between the mild, moderate and severe spasticity level groups using the described classification, and they were retrospectively found to fit the overall clinical picture; none of the children in the SDR group ended up in the mild spasticity level group at baseline.

### GMFCS

The GMFCS levels were classified after and as close to the fourth birthday as possible, and they were used to stratify the study population at baseline. At 4 years of age, the GMFCS level, CP diagnosis, and CP subtype can be decided with high or acceptable accuracy [25, 40].

Retrospective, although not psychometrically tested, classification of GMFCS levels based on clinical descriptions in medical records was used in the 2002 metanalysis of the three North American SDR RCTs [35]. In the present study, structured data from the CPUP registry on functional performance and capability was available for retrospective classification of GMFCS levels at baseline date before the GMFCS was introduced. The kappa analysis of this classification showed good agreement (κ = 0.732, *p* < 0.001), as described in Additional file 1, and the oldest birth year cohorts could be included in the study.

To be able to use the GMFCS level in the logistic regression, the levels were dichotomized into levels I-II and III-V. According to Hägglund et al. [17], scoliosis mainly appear in GMFCS III-V, and very seldom in GMFCS I-II and thus the subgroups I-II vs III-V make clinical sense.

### Scoliosis

The multilevel laminoplasty technique used to access the rootlets for the SDR procedure in the present study included reinstatement of laminae and did not increase the occurrence of scoliosis after SDR. For the SDR GMFCS IV-group, scoliosis occurred even to a lesser extent and with later onset than for GMFCS IV-control group (Fig. 3c). Development of contractures and asymmetries, especially common in higher GMFCS-levels [17, 41, 42], may be less severe after SDR combined with physiotherapy, as use of orthoses, sitting and supported standing positions with more symmetric spine may be more easy to obtain after tonus reduction.

In the logistic regression analysis of variables to explain scoliosis or not in the whole group at 20 years of age, the variable *GMFCS* contributed significantly. The other variables; SDR surgery, spasticity level at base-line, and sex did not contribute to explain scoliosis at 20 years of age. Several studies have previously shown that girls are more affected by scoliosis than boys [16, 17]. However, in the present study children with contraindications to SDR, such as ataxic or dyskinetic traits were excluded, obstructing comparisons.

In SDR, other forms of spinal misalignments, especially spondylolisthesis have been reported more frequent than in the general population [6, 7, 9, 11, 12], even if scoliosis was reported to be the most common deformity following SDR [4]. Studies reporting spinal deformities after SDR are not population based and with no or small comparison groups [4]. To further explore spinal misalignments after SDR in the present population, results regarding imaging of the spine beyond scoliosis and Cobb angles would be needed. Absence of spinal pain may, however, indicate absence of significant spinal problems.

### Pain

Pain in the CP population has previously not been properly noticed [33], even if it is one of the most common co-morbidities [43]. Beside no increase in scoliosis development after SDR, the other main finding of the present study was that the frequency of spinal pain did not differ between the SDR group and the control group at 10, 15, 20 and 25 years of age (Table 3).

### Limitations

Only variables already included in the CPUP registry were available, which limits the research. However, a major strength of the registry data available from a total Swedish population of individuals with CP continuously, collected during the last 25 years.

The low proportion of individuals born 1990–1997, available to serve as natural history comparison group in adult age, is a limitation (Table 1). The control group thus included a higher proportion of younger persons, who probably received somewhat different care compared to the older cohorts [19, 20]. In study participants born 1994 and later, in contrast to those born 1990–1993, some were treated with botulinum toxin with a lowered muscle tone at the baseline assessment.

Assessments and registrations were performed regularly using a standardized methodology by clinicians in their daily practice, and limited information was available, as only the most important items can be included to keep a register acceptably time-consuming. The spinal screening lacked information on other misalignments than scoliosis. Cobb angles were inconsistently registered at the time data was extracted from the register, but have been completed later, available for future studies.

The late introduction of pain screening in the registry resulted in low numbers of recorded answers about spinal pain at different ages, which is another limitation. There were slightly more frequent recordings of spinal pain in the SDR than in the control group, although the differences were not statistically significant. Although possible reduction of pain in adults with CP after early SDR is reported [44], the authors are anxious to find out whether SDR at a young age is causing more spinal pain than expected from natural history. Information of pain intensity, duration, effect on daily living or quality of life was available only in the adult age CPUP forms.

Spinal pain several years after surgical correction of scoliosis, as described in the present study, was found also in a population based study with high number of participants [16]. Increased awareness among health professionals of the importance of pain assessments in this population led to extended pain questions in the most recent version of the CPUP PT-form, so more and higher quality data will be available in the future.

Due to the relatively small numbers of individuals with scoliosis and spinal pain, more simple statistical methods were used for the analyses, and the data allowed only few variables to be included in the logistic regression models.

### Generalizability

This study represents the real-life situation in the ordinary health care, in contrast to RCTs, or other experimental study designs, usually conducted at tertial health care level after rigorous selection of participants. The total population with CP in certain age cohorts were included in the present study, without selection bias. Results would be generalizable in populations where the socio-economic and health care standards are comparable to those in Sweden. Also, the surgery technique, multi-level SDR without permanent removal of the spinal laminae/spinous processes, was used for all individuals in the study, and is commonly used internationally.

## Conclusion

This population based longitudinal matched outcome study, provides evidence against long-term complications from the spine caused by the SDR surgery. Individuals undergoing SDR had similar development of scoliosis as comparable controls. In addition, individuals with most functional limitations, GMFCS IV, who had SDR in young age had later onset and lower occurrence of scoliosis than their peers in the non-SDR group. GMFCS was the variable that best could explain scoliosis at 20 years of age; SDR surgery, sex or base-line spasticity level did not. Spinal pain was reported at similar levels for SDR operated and controls up to the age of 25 years.

## Supplementary Information

**Additional file 1: ** Retrospectively performed Gross Motor Function Classification System (GMFCS)-assessments using other Cerebral Palsy follow-UP registry (CPUP) data of gross motor function. **Table Appendix.** Retrospectively assessed GMFCS levels by researcher using CPUP data, compared to assessment by local physiotherapis.

## Data Availability

Data used in this study are stored at the National Quality Registry CPUP (http://rcsyd.se/anslutna-register/cpup). Data are not publicly available and permission to extract data can be obtained from the registry holder on reasonable request. Information on variables used for the present study is available from the authors.

## Abbreviations

CP: Cerebral palsy
BSCP: Bilateral spastic CP
SDR: Selective Dorsal Rhizotomy
GMFCS: Gross Motor Functions Classification System
ITB: Intrathecal Baclofen
CPUP: The Swedish national secondary prevention follow-up program in cerebral palsy
PT: Physiotherapy
MAS: Modified Ashworth Scale
SCPE: Surveillance of Cerebral Palsy in Europe
