# Supplementary material for: No support that early selective dorsal rhizotomy increase frequency of scoliosis and spinal pain – a longitudinal population-based register study from four to 25 years of age
Source: BMC Musculoskelet Disord. 2020 Nov 27;21:782. doi: 10.1186/s12891-020-03782-5 (PMC7697382; doi:10.1186/s12891-020-03782-5)
Supplement: Supplementary file 1 — Additional file 1: Retrospectively performed Gross Motor Function Classification System (GMFCS)-assessments using other Cerebral Palsy follow-UP registry (CPUP) data of gross motor function. Table Appendix. Retrospectively assessed GMFCS levels by researcher using CPUP data, compared to assessment by local physiotherapis. [file 12891_2020_3782_MOESM1_ESM.docx]

*Retrospectively performed Gross Motor Function Classification System (GMFCS)-assessments using other Cerebral Palsy follow-UP registry (CPUP) data of gross motor function*

CPUP registry variables describing gross motor activities in daily life were used to test retrospective classification of GMFCS levels (Palisano et al. 2008). All children with bilateral spastic CP (BSCP) born 1991-2002, who had a GMFCS recorded by the child´s physiotherapist were included (n=126). Author LW prepared a file from the first CPUP assessment after the child´s fourth birthday or the last before SDR. Median age at assessment was 4.25 years (interdecile range 4.00-4.99 years, interquartile range 4.08-4.50, and total range 2.67-10.0 years). All names, personal identification numbers, and reported GMFCS levels were removed. No variables other than the descriptions of different gross motor activities and “Wilsons gait scale” (referred to in Tedroff et al. 2015) were included. Author ALJ classified GMFCS level according to the original GMFCS manual (Palisano et al. 1997) using these de-identified data on motor function in the standardised CPUP form (http://cpup.se/sjukgymnastik-barn/).

To check the reliability of these retrospectively assessed GMFCS levels from other data in the CPUP reports, a kappa agreement test was performed with GMFCS classified by the child’s local physiotherapist at assessment of the same children/reports. The agreement was κ=0.732 (p-value <0.001), and retrospective assessment based on other CPUP variables made by ALJ was considered as useful in the further analysis.

Table Appendix. Retrospectively assessed GMFCS levels by researcher using CPUP data, compared to assessment by local physiotherapist

|  | Retrospective assessment based on CPUP variables | | | | |  |
| --- | --- | --- | --- | --- | --- | --- |
| Clinical assessment | GMFCS I | GMFCS II | GMFCS III | GMFCS IV | GMFCS V | Total |
| GMFCS I | 40 | 2 | 0 | 0 | 0 | 42 |
| GMFCS II | 5 | 13 | 1 | 0 | 0 | 19 |
| GMFCS III | 0 | 4 | 19 | 8 | 0 | 31 |
| GMFCS IV | 0 | 0 | 0 | 18 | 2 | 20 |
| GMFCS V | 0 | 0 | 0 | 4 | 10 | 14 |
| Total | 45 | 19 | 20 | 30 | 12 | 126 |

Legend: GMFCS: Gross Motor Function Classification System, CPUP: the Swedish national secondary prevention follow-up program in cerebral palsy.

**References:**

Rosenbaum PL, Palisano RJ, Bartlett DJ, Galuppi BE, Russell DJ. Development of the Gross Motor Function Classification System for cerebral Palst. Dev Med Child Neurol. 2008: 50(4):249-53.

Tedroff K, Lowing K, Astrom E. A prospective cohort study investigating gross motor function, pain, and health-related quality of life 17 years after selective dorsal rhizotomy in cerebral palsy. Dev Med Child Neurol. 2015; 57(5): 484-90

Palisano R, Rosanbaum P, Walter S, Russell D, Wood E, Galuppi B. Development and reliability of a system to classify gross motor function in children with cerebral palsy: a retrospective cohort registry study. Dev Med Child Neurol. 1997; 39(4):214-23.
